# Assessing thermal adaptation of a global sample of *Aspergillus fumigatus*: Implications for climate change effects

**DOI:** 10.3389/fpubh.2023.1059238

**Published:** 2023-02-16

**Authors:** Greg Korfanty, Erin Heifetz, Jianping Xu

**Affiliations:** Department of Biology, McMaster University, Hamilton, ON, Canada

**Keywords:** human fungal pathogen, temperature effect, reaction norm, heritability, microsatellite genotyping, coefficient of variation, geographic population

## Abstract

*Aspergillus fumigatus* is a common environmental mold and a major cause of opportunistic infections in humans. It's distributed among many ecological niches across the globe. A major virulence factor of *A. fumigatus* is its ability to grow at high temperature. However, at present, little is known about variations among strains in their growth at different temperatures and how their geographic origins may impact such variations. In this study, we analyzed 89 strains from 12 countries (Cameroon, Canada, China, Costa Rica, France, India, Iceland, Ireland, New Zealand, Peru, Saudi Arabia, and USA) representing diverse geographic locations and temperature environments. Each strain was grown at four temperatures and genotyped at nine microsatellite loci. Our analyses revealed a range of growth profiles, with significant variations among strains within individual geographic populations in their growths across the temperatures. No statistically significant association was observed between strain genotypes and their thermal growth profiles. Similarly geographic separation contributed little to differences in thermal adaptations among strains and populations. The combined analyses among genotypes and growth rates at different temperatures in the global sample suggest that most natural populations of *A. fumigatus* are capable of rapid adaptation to temperature changes. We discuss the implications of our results to the evolution and epidemiology of *A. fumigatus* under increasing climate change.

## 1. Introduction

Among the vast diversity of biotic and abiotic factors that can impact the evolution of organisms, temperature has undoubtedly captured more than its share of attention. Biologists have linked variations in temperature to everything from temporal patterns of growth, survival, and reproduction of individual organisms to broad spatial patterns of population density and species distributions across a range of geographic scales. These studies have shown that the same change in temperature often affect different organisms differently. Even for the same organism, temperature does not affect all life stages equally. However, most thermal adaptation studies so far have focused on plants and animals. Relatively little is known about how temperature impact the spatial and temporal distributions of microbial populations, including populations of human fungal pathogens. With increasing climate change and global warming, there is a pressing need to understand the thermal adaptation of these organisms in order to better prepare for potential future epidemics and pandemics (1).

The ascomycete mold *Aspergillus fumigatus* is among the most common opportunistic human pathogens (1, 2). It's ubiquitously distributed in a diversity of ecological niches such as air, water, compost, and soil across the globe. *A. fumigatus* can cause a broad spectrum of opportunistic infections collectively termed aspergillosis that affects approximately 8 million individuals worldwide (3). Among aspergillosis infections, invasive aspergillosis is the most severe, responsible for ~250,000 deaths worldwide each year (1–3). At risk-populations include immunocompromised patients such as patients receiving immunosuppressive drugs due to haematopoietic stem cell or solid organ transplants, patients with severe neutropenia, and as co-infections in patients with coronavirus disease 2019 (COVID-19) (2, 4). Different from other human fungal pathogens, *A. fumigatus* can grow at temperatures above 50°C (3). However, the selective forces and genetic mechanisms govern its high thermotolerance remains largely unknown.

Population genetic surveys have shown that the global population of *A. fumigatus* consists of at least three distinct clades and multiple genetic clusters (5–7). These genetic clusters were likely historically differentiated from each other due to geographic separations. However, evidence for gene flow and clonal dispersal have been found across countries and continents, resulting in most regional geographic populations containing strains of different clades and different genetic clusters (5, 8–10). Some of these dispersals were most likely driven by contemporary anthropogenic activities, including human travel and commercial trade (8, 10). In addition, agricultural fungicides are creating significant selective pressure where drug-resistant strains are rapidly spreading (11). The identification of successful recent migrants across broad geographic scales and ecological niches with different temperature spectra suggests that there might be little or no variation in thermal adaptation among strains of *A. fumigatus*. Alternatively, the individual genomes of *A. fumigatus* may have high adaptive potential and/or that immigrants may be readily recombining with local strains to acquire a thermal response profile adapted to local environments. At present, little is known about the geographic and ecological patterns of thermal adaptation of *A. fumigatus* populations.

With the effects of climate change in a diversity of areas being increasingly felt, an important question to consider is how climate change may affect natural *A. fumigatus* populations in different areas around the world. With increasing temperatures, potentially more thermotolerant strains will likely emerge and that *A. fumigatus* may further expand to colder regions. Indeed, global warming is the major suspected cause of the newly emerged and highly antifungal resistant pathogen *Candida auris* that has already caused many outbreaks across the world (12–14). The emergence of *C. auris* occurred simultaneously across multiple continents and is hypothesized to be the first documented occurrence of a pathogen emerging due to global warming (15, 16).

In this study, we aim to characterize the variations among strains in their growths at different temperatures and investigate how their geographic origins and genetic relationships may impact such variations. To achieve the goals, we selected strains that originated from 12 countries. The phenotypic plasticity of these strains to temperature, which we termed as strain thermal adaptation, was measured *via* growth in liquid media at four incubation temperatures. Elucidating the impact of temperature on strain growth variation will provide insights on how *A. fumigatus* may adapt to global climate change. Such knowledge should help us develop better understanding of *A. fumigatus* epidemiology and minimize the disease burden on humans.

## 2. Methodology

### 2.1. Geographic populations of *A. fumigatus*

In total, 89 *A. fumigatus* strains were analyzed in this study. These strains were obtained from a variety of ecological niches and geographic locations (Table 1). They were selected from our strain collections to represent different mating types, antifungal susceptibilities, and ecological and geographic origins. Among the 89 strains, 77 were isolated from soil samples collected from 11 countries: Cameroon (11 strains), Canada [Northwest Territories (NWT) (3 strains) and Hamilton, Ontario (11 strains)], China (10 strains), Costa Rica (6 strains), France (3 strains), India (4 strains), Iceland (3 strains), Ireland (1 strains), New Zealand (11 strains), Peru (3 strains), and Saudi Arabia (11 strains). Environmental strains from Cameroon, Canada, China, Costa Rica, France, Iceland, New Zealand, Peru and Saudi Arabia have been described in previous studies (5, 8, 10). Environmental strains from soil samples were isolated following the procedures described in Samarasinghe et al. (17). The remaining 12 strains were from patients in Hamilton, Ontario (5 strains), New Delhi, India (6 strains), and the US (1 strain) (18, 19). Two strains, one from Ireland (environmental) and the other from US (clinical), represented two super-maters of this species and they were included in our study (20). For additional strain information, please refer to Supplementary Table S1.

**Table 1:** Metadata about the 89 *A. fumigatus* strains used in this study.

**Strain ID**	**Country**	**Region**	**Average temperature (°C)**	**Temperature range of monthly averages (°C)**	**Latitude**	**Longitude**	**Source**
C44	Cameroon	Mbingo	19.9	26.8 to 13.1	6.164	10.290	Environ.
C65	Cameroon	Bambui	19.9	26.8 to 13.1	6.015	10.232	Environ.
C79	Cameroon	Bambui	19.9	26.8 to 13.1	6.015	10.232	Environ.
C158	Cameroon	Makepe	26	30 to 23	4.064	9.741	Environ.
C304	Cameroon	Eloundem	23	29 to 18	3.838	11.437	Environ.
C308	Cameroon	Eloundem	23	29 to 18	3.838	11.437	Environ.
C322	Cameroon	Eloundem	23	29 to 18	3.838	11.437	Environ.
C372	Cameroon	Mbalgong	23	29 to 18	3.803	11.468	Environ.
C428	Cameroon	Simbock	23	29 to 18	3.821	11.475	Environ.
C443	Cameroon	Simbock	23	29 to 18	3.821	11.475	Environ.
C480	Cameroon	Mbandoumou	23	29 to 18	3.791	11.453	Environ.
AC10-2	China	Ailao mountains	14	25 to 2	24.206	101.370	Environ.
AC3-3	China	Ailao mountains	14	25 to 2	24.206	101.370	Environ.
AC3-4	China	Ailao mountains	14	25 to 2	24.206	101.370	Environ.
AC6-5	China	Ailao mountains	14	25 to 2	24.206	101.370	Environ.
AC7-1	China	Ailao mountains	14	25 to 2	24.206	101.370	Environ.
AC7-10	China	Ailao mountains	14	25 to 2	24.206	101.370	Environ.
C2-5	China	Fenyi	18.2	29.9 to 2.2	27.828	114.687	Environ.
C4-1	China	Fenyi	18.2	29.9 to 2.2	27.828	114.687	Environ.
C4-2	China	Fenyi	18.2	29.9 to 2.2	27.828	114.687	Environ.
C5-10	China	Fenyi	18.2	29.9 to 2.2	27.828	114.687	Environ.
EJ13	Costa Rica	El Jardin	21	30.2 to 18.2	10.016	−84.214	Environ.
EJ24	Costa Rica	El Jardin	21	30.2 to 18.2	10.016	−84.214	Environ.
MA28	Costa Rica	Manuel Antonio	25.8	33.4 to 19.1	9.392	−84.137	Environ.
MA31	Costa Rica	Manuel Antonio	25.8	33.4 to 19.1	9.392	−84.137	Environ.
LF18	Costa Rica	La Fotuna	21	30.2 to 18.2	10.468	−84.643	Environ.
LF25	Costa Rica	La Fotuna	21	30.2 to 18.2	10.468	−84.643	Environ.
DF29	France	Downtown Nice	16	27.7 to 5.3	43.710	7.262	Environ.
TF59	France	Oldtown Nice	16	27.7 to 5.3	43.710	7.262	Environ.
HF16	France	Hyeres	15.3	28.2 to 4.3	43.121	6.129	Environ.
AV88	Canada	St. George	7.9	26.5 to −9.3	43.271	−80.250	Environ.
CF10	Canada	St. George	7.9	26.5 to −9.3	43.271	−80.250	Environ.
CM11	Canada	St. George	7.9	26.5 to −9.3	43.271	−80.250	Environ.
CM16	Canada	St. George	7.9	26.5 to −9.3	43.271	−80.250	Environ.
CM21	Canada	St. George	7.9	26.5 to −9.3	43.271	−80.250	Environ.
CM38	Canada	St. George	7.9	26.5 to −9.3	43.271	−80.250	Environ.
CM58	Canada	St. George	7.9	26.5 to −9.3	43.271	−80.250	Environ.
15-1	Canada	Hamilton	7.9	26.5 to −9.3	43.263	−79.856	Clinical
15-09	Canada	Hamilton	7.9	26.5 to −9.3	43.263	−79.856	Clinical
15-21	Canada	Hamilton	7.9	26.5 to −9.3	43.263	−79.856	Clinical
15-34	Canada	Hamilton	7.9	26.5 to −9.3	43.263	−79.856	Clinical
15-42	Canada	Hamilton	7.9	26.5 to −9.3	43.263	−79.856	Clinical
M14	Canada	Hamilton	7.9	26.5 to −9.3	43.261	−79.919	Environ.
M16	Canada	Hamilton	7.9	26.5 to −9.3	43.261	−79.919	Environ.
P20	Canada	Hamilton	7.9	26.5 to −9.3	43.277	−79.786	Environ.
P80	Canada	Hamilton	7.9	26.5 to −9.3	43.277	−79.786	Environ.
T34	Iceland	Thingvellir	4	12 to −2	64.256	−21.130	Environ.
SF37	Iceland	Skaftafell	4	13 to −3	64.070	−16.975	Environ.
N9	Iceland	Nautholsvik	4	12 to −2	64.124	−21.927	Environ.
I1268	India	New Delhi	25	40.5 to 6.7	28.598	77.222	Clinical
I1272	India	New Delhi	25	40.5 to 6.7	28.598	77.222	Clinical
I1591	India	New Delhi	25	40.5 to 6.7	28.598	77.222	Clinical
I162	India	New Delhi	25	40.5 to 6.7	28.598	77.222	Environ.
I245	India	New Delhi	25	40.5 to 6.7	28.598	77.222	Clinical
I2581	India	New Delhi	25	40.5 to 6.7	28.598	77.222	Clinical
I384	India	New Delhi	25	40.5 to 6.7	28.598	77.222	Environ.
I388	India	New Delhi	25	40.5 to 6.7	28.598	77.222	Environ.
I437	India	New Delhi	25	40.5 to 6.7	28.598	77.222	Environ.
I591	India	New Delhi	25	40.5 to 6.7	28.598	77.222	Clinical
AFIR928	Ireland	Dublin	9.8	19.5 to 2.3	−6.313	53.324	Environ.
A3-4	New Zealand	Trusts Arena	15.5	23.6 to 7.5	−36.866	174.636	Environ.
A6-6	New Zealand	Trusts Arena	15.5	23.6 to 7.5	−36.866	174.636	Environ.
D2-6	New Zealand	Aukland	15.5	23.6 to 7.5	−36.859	174.776	Environ.
D6-5	New Zealand	Auckland Domain	15.5	23.6 to 7.5	−36.859	174.776	Environ.
M3-8	New Zealand	Millenium Field	15.5	23.6 to 7.5	−36.743	174.731	Environ.
M4-8	New Zealand	Millenium Field	15.5	23.6 to 7.5	−36.743	174.731	Environ.
R5-6	New Zealand	Aukland Rail	15.5	23.6 to 7.5	−36.849	174.765	Environ.
U5-1	New Zealand	Auckland U.	15.5	23.6 to 7.5	−36.850	174.770	Environ.
V1-8	New Zealand	Mount Eden	15.5	23.6 to 7.5	−36.877	174.765	Environ.
V6-1	New Zealand	Mount Eden	15.5	23.6 to 7.5	−36.877	174.765	Environ.
V8-8	New Zealand	Mount Eden	15.5	23.6 to 7.5	−36.877	174.765	Environ.
1_18	Canada	Yellowknife, NWT	−4.3	21.3 to −29.5	62.454	−114.372	Environ.
5_4	Canada	Yellowknife, NWT	−4.3	21.3 to −29.5	62.454	−114.372	Environ.
6_13_2	Canada	Yellowknife, NWT	−4.3	21.3 to −29.5	62.454	−114.372	Environ.
RM7-10	Peru	Rainbow Mountain	12	20 to 1	−13.618	−71.844	Environ.
LP19-2	Peru	Lima	20	26 to 15	−12.046	−77.043	Environ.
SV28-4	Peru	Sacred Valley	12	20 to 1	−13.333	−72.085	Environ.
AML22	Saudi Arabia	Al-Madina East	27	38 to 12	24.473	39.610	Environ.
AML81	Saudi Arabia	Al-Madina East	27	38 to 12	24.473	39.610	Environ.
Jed22	Saudi Arabia	Jeddah	28	37 to 18	21.606	39.171	Environ.
Jed47	Saudi Arabia	Jeddah	28	37 to 18	21.606	39.171	Environ.
Jed57	Saudi Arabia	Jeddah	28	37 to 18	21.606	39.171	Environ.
Jed70	Saudi Arabia	Jeddah	28	37 to 18	21.606	39.171	Environ.
Jed71	Saudi Arabia	Jeddah	28	37 to 18	21.606	39.171	Environ.
Jed75	Saudi Arabia	Jeddah	28	37 to 18	21.606	39.171	Environ.
AML38	Saudi Arabia	Al-Madina East	27	38 to 12	24.525	39.569	Environ.
Yan179	Saudi Arabia	Yanbu	27	37 to 15	24.088	38.067	Environ.
Yan67	Saudi Arabia	Yanbu	27	37 to 15	24.088	38.067	Environ.
AFB62-1	United States	San Antonio	20.6	34.9 to 4.3	29.425	−98.492	Clinical

Average temperature and temperature range data at each site were extracted from Weatherbase.com.

### 2.2. Experimental conditions

To identify variations in growth among *A. fumigatus* strains at different temperatures, strains were grown at the following five temperatures in triplicates per temperature, 4°C, 15°C, 22°C, 35°C, and 41°C. For each strain, an inoculum was created and used to assess growth at all temperatures. To prepare the inoculum, *A. fumigatus* strains were cultured on malt extract agar (MEA) for 2–3 days at 37°C. Conidia were then harvested by dispensing 1 ml of a sterile 0.85% saline solution onto the culture and aspirating the conidial suspension to a sterile 1.5 ml tube. Conidial density was measured using a Countess^®^ II FL automated cell counter and adjusted to 1 × 10^8^ conidia/ml in saline. Conidial suspensions were diluted in RPMI 1,640 to a concentration of 1 × 10^6^ conidia/ml. In total 200 μL of this suspension was then aliquoted in triplicates into a 96 well microtiter plate for each temperature. Relative growth was estimated using the optical density (OD) values at 600 nm absorbance using the Biotek Epoch™ 2 Microplate Spectrophotometer. Growth measurements were taken at four time points: immediately after inoculation, and 24 h, 48 h, and 72 h post inoculation. For each 96-well microtiter plate, three wells with medium but without any fungal culture were used as negative controls.

### 2.3. Strain genotyping and geographic climate data

Each *A. fumigatus* strain was genotyped at nine highly polymorphic short tandem repeat (STR) loci (also called microsatellite loci) and at the mating type (MAT) locus. Strain genotyping at the nine STR loci followed the protocol described by De Valk et al. (21). The mating type of each strain was identified following the protocol described by Paoletti et al. (22).

Atmospheric temperature profiles of the geographical locations where individual strains were isolated were obtained using the website Weatherbase (weatherbase.com). Specifically, the closest city to the sampling site with recorded data was used as a proxy of the temperature at each location. The average temperature, highest and lowest monthly average temperature across all months of a year, and the temperature range between the highest and lowest monthly average temperatures were collected.

### 2.4. Statistical analysis

The growth of each strain under each temperature condition was obtained at three time points. To identity differences in growth between strains and populations, pairwise *t*-tests were conducted. *Post hoc* corrections followed the Holm method. No growth was observed for any strain at 4°C. Thus, our analyses of growths were conducted on data at the remaining four temperatures 15°C, 22°C, 35°C, and 41°C. All analyses were conducted in R version 4.2.1 (23). Specifically, the following analyses were conducted.

In the first, we constructed reaction norm plots for all strains across four temperatures to show if there are strain x temperature interactions. The mean OD value of each strain at each temperature was used to construct the reaction norm plot.

Second, the broad sense heritability contributing to growth differences among strains was calculated using the below formula.


$$H^{2}\;=\frac{V_{G}}{V_{P}}=\;\frac{\left(V_{P}-V_{E}\right)}{V_{P}}$$

where *H*^2^ is the broad sense heritability, *V*_*P*_ is the phenotypic variance calculated as the total variance in growth across all strains, and *V*_*G*_ is the genotypic variance, calculated as the difference between *V*_*P*_ and environmental variance (*V*_*E*_). *V*_*E*_ is calculated as the average of the variances in growth between replicates over all strains. *H*^2^ was calculated for each temperature on each of the 3 days.

Third, a mixed ANOVA was conducted to determine the contribution of country, temperature, and day post incubation on strain growth. The r package *rstatix* was used to conduct the ANOVA.

Forth, for each strain, we quantified the extent of variation in their growths among temperatures, using the measure of coefficient of variation. The coefficient of variation (CV), shown below,


$$\begin{array}{l} CV=\;\frac{\sigma }{\mu } \end{array}$$

is the ratio of standard deviation (σ) to the mean (μ). A large CV represents big differences in growth rates among temperatures. In contrast, a small CV represents relative uniformity in growths among temperatures for the specific strain. The CV was calculated for each *A. fumigatus* strain using growth values across all four temperatures.

Fifth, the contributions of mating type, temperature range and average temperature where strain came from on the CV of each strain on each day were estimated as follows. For each independent variable, the assumptions of normality were verified using Shapiro–Wilk's test and homoscedasticity using the Breusch–Pagan test. For mating type, we used both the parametric Student's *T*-test and the non-parametric Mann-Whitney test to compare the differences between them. For temperature range and average temperature, a linear regression model was generated to determine significance in the relationship between the temperature parameters in their native environments on CV.

Lastly, we tested whether the difference in CV among strains were related to their genotypic relationships as determined based on the nine STR loci. Specifically, we obtained two matrices and conducted non-parametric Mantel test between them. In one matrix, we calculated the absolute difference in CV between all pairs of strains. In the other matrix, Bruvo's genetic distance between all pairwise combination of strains was calculated using *bruvo.dist* in the R package *poppr* (24). Bruvo's genetic distance is specific for STR loci and it incorporates the stepwise mutation model during genetic distance calculations. A Shapiro-Wilk's test was conducted on the residuals to determine whether the distributions of the two matrices were normal. A linear regression model was used to determine the relationship between differences in genetic distance and in CV between pairs of strains. All 3,916 pairwise strain combinations were included in the analysis. Additionally, using the Bruvo's genetic distance matrix, a neighbor joining tree was generated using the R package *ape* (25). The tree was edited and visualized through the Interactive Tree of Life (iTOL) website (26).

## 3. Results

### 3.1. Growth is significantly influenced by temperature but remains highly varied within four of the five tested temperatures

**Figure 1:**
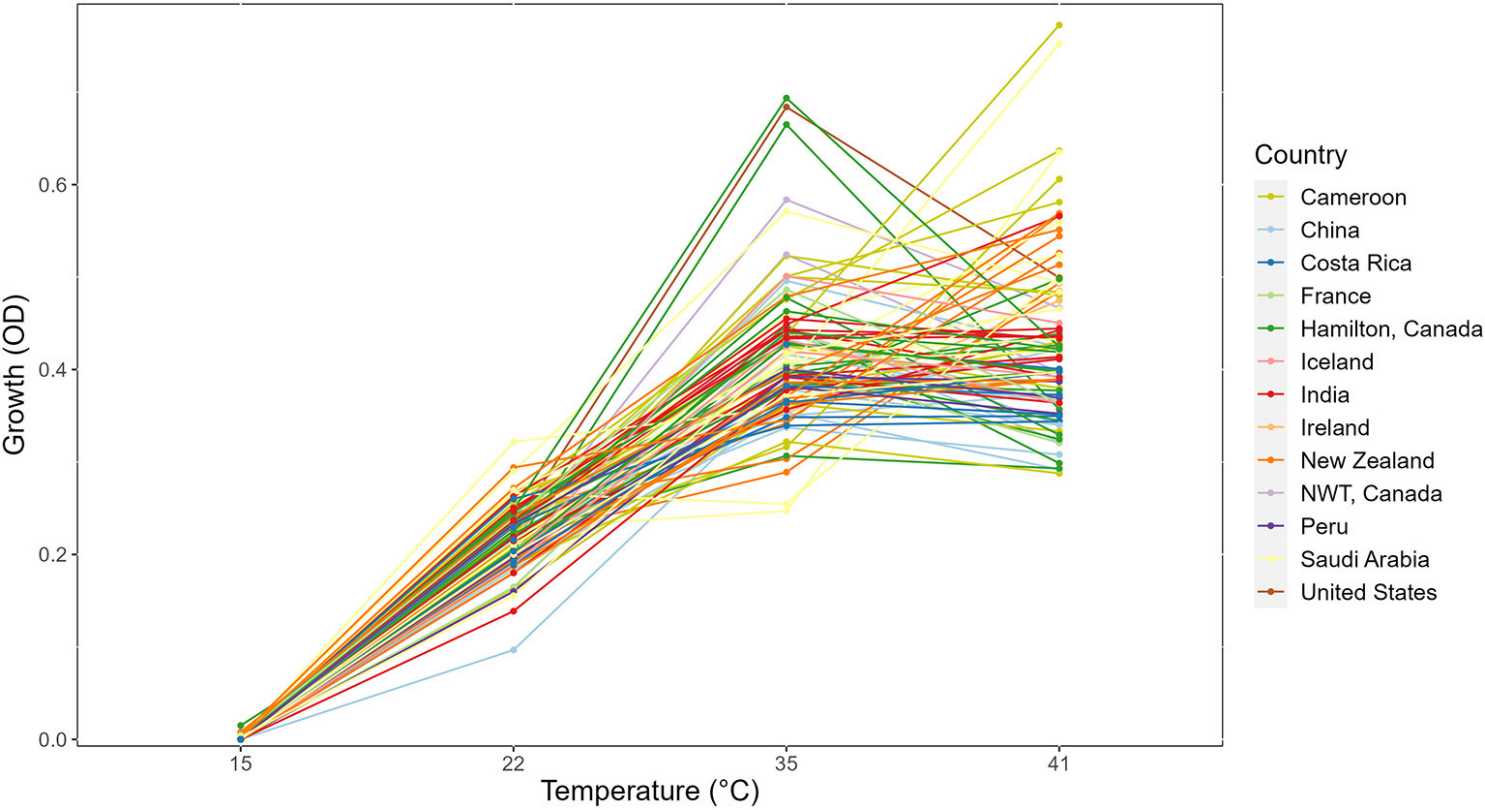
Reaction norm plot showing strain × temperature interactions in the day 3 growth profiles of 89 strains across four temperatures. NWT, Northwest Territories, Canada.

In this study, we determined the growths of 89 strains for 3 days at 5 different temperatures. No growth was found at the 4°C environment for any of the 89 strains. Thus, our analyses will be focused on the remaining four temperatures. The growth profiles for all strains at the four temperatures are shown in Figure 1 as the reaction norm plots. For better visualization and comparison among strains within each country, growth profiles of strains separated by country of origin were also generated (Supplementary Figure 1). Among the four temperatures, significant growth differences between temperatures were observed (Figure 1). Overall, among these four temperatures, limited growths were observed at 15°C for most strains over all 3 days. Similarly, there was limited growth within 24 h at 22°C. However, broad variations among strains were observed at both the 35°C and 41°C environments. Interestingly, many strains showed similar growths at 35°C and 41°C.

To investigate how much of the differences in growth at each temperature at each of the three time points were due to genetic differences among strains, we calculated the broad sense heritability (BSH) of the growth observed among our strains (Table 2). Our analyses revealed that in 10 of the 12 temperature × date combinations, their BSH values were all greater than 0.5. The only two combinations with BSH <0.5 were growths at 35°C during day 2 and day 3. Overall, at each of the 3 days, the 35°C environment showed the lowest BSH while the 22°C and 41°C showed the highest. However, there was no obvious pattern among the 3 days at each of the four temperatures.

**Table 2:** Broad sense heritability (BSH, H^2^) calcuated using the observed growth of the 89 *A. fumigatus* strains across the 3 days and four temperatures.

**Day**	**Temperature**			
	**15**°**C**	**22**°**C**	**35**°**C**	**41**°**C**
24 h	0.454	0.675	0.514	0.749
48 h	0.654	0.806	0.368	0.565
72 h	0.652	0.703	0.355	0.701

**Figure 2:**
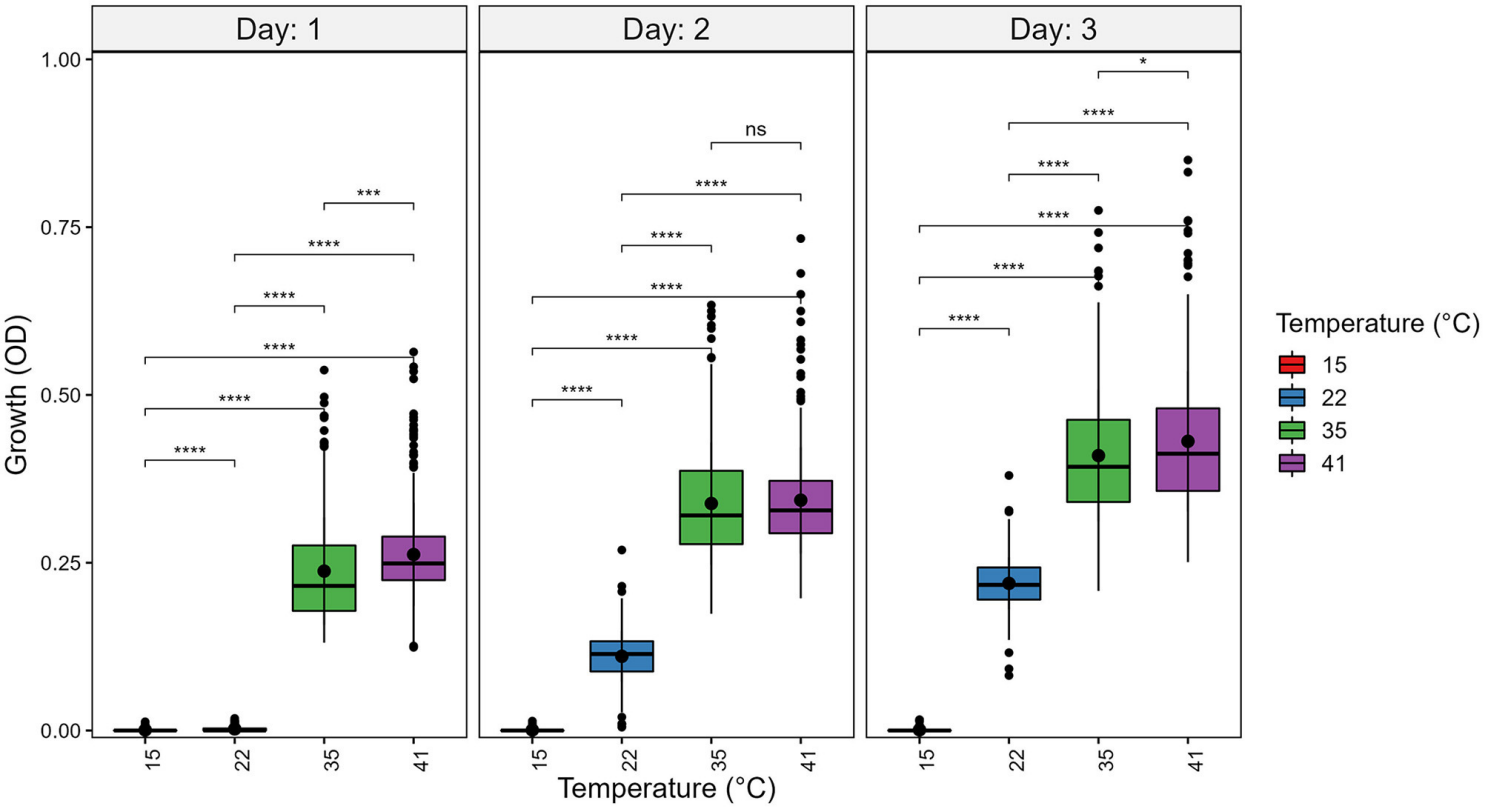
Temperature significantly contributes to growth differences among strains of *A. fumigatus*. *A. fumigatus* strains were grown at four temperatures: 15°C, 22°C, 35°C, and 41°C during a 3-day period. To quantify growth, the optical density (OD) at 600 nm of each strain was measured. Significance was determined through pairwise *t*-tests with *post hoc* correction *via* Holm's method. Boxplot center line represent the median and the top and bottom represent interquartile range. *p-value < 0.05, ***p-value < 0.001, ****p-value < 0.0001.

**Figure 3:**
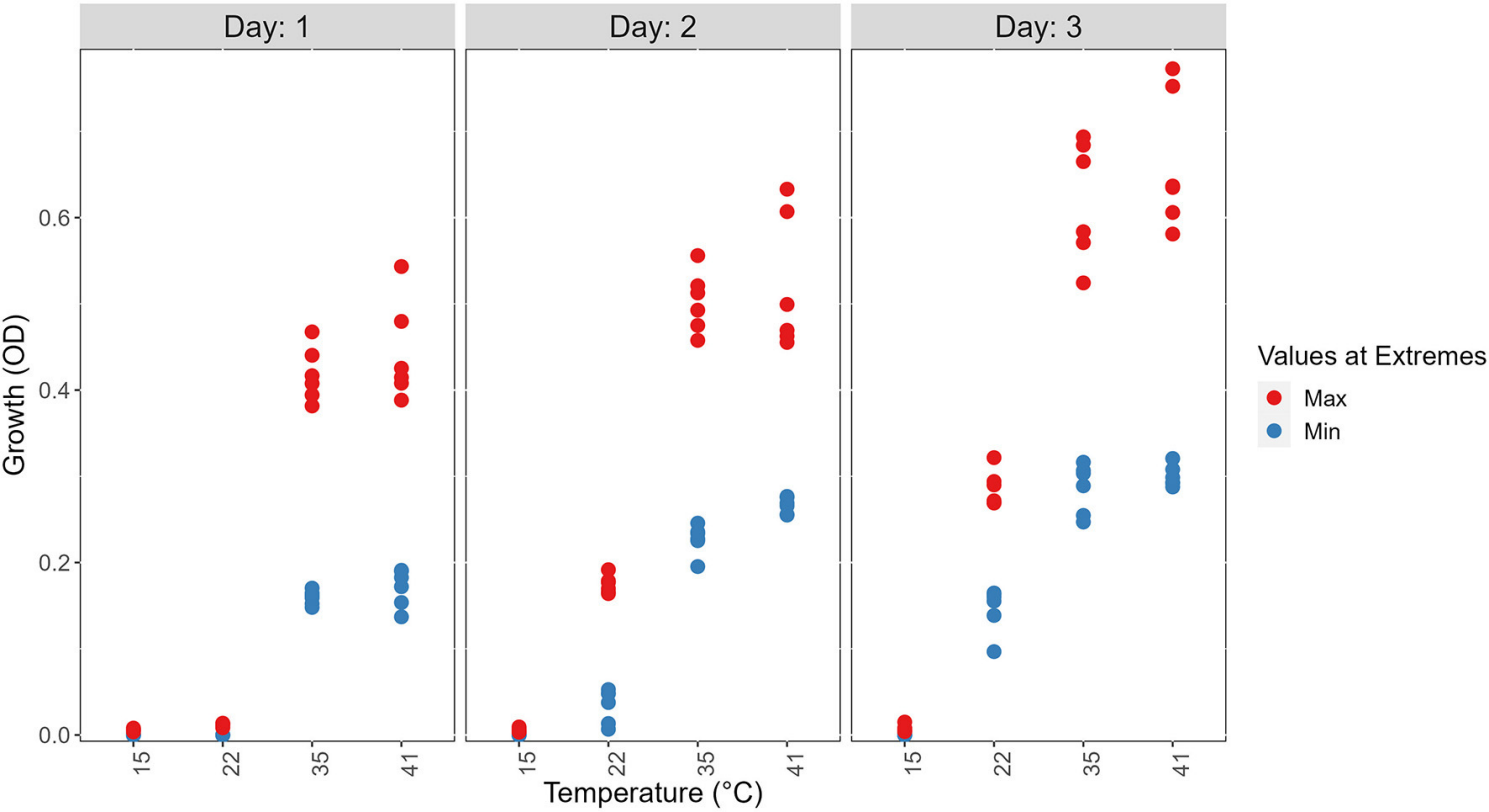
Visual representations of the top six fastest growing strains and the bottom six slowest growing ones at each of the four temperatures on 3 days to show the range of growth difference of *A. fumigatus* strains. The top 6 (Max) are shown in red and the bottom 6 (Min) are shown in blue.

**Figure 4:**
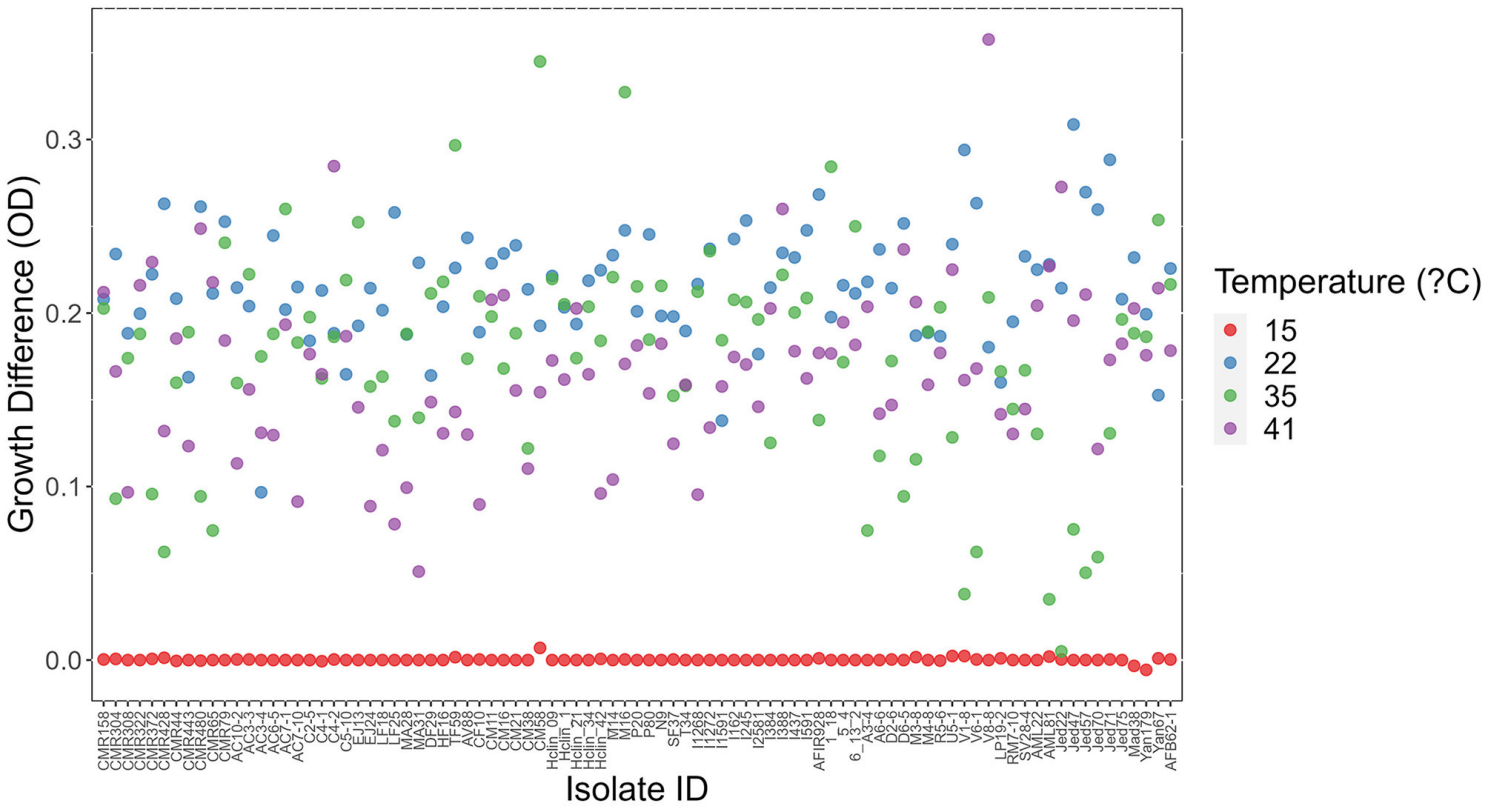
Difference in growth between day 3 and day 1 at each of four temperatures among 89 *A. fumigatus* strains. X-axis represents strain names. Y-axis represents in OD value differences between day 3 and day 1.

At each temperature, we observed substantial variations in growth among strains (Figures 1, 2). As the temperature increases, the range of growth rates became wider among strains. Similarly, as time progresses, the growth differences among most strains became more obvious and the standard deviations correspondingly increased. To highlight some of the differences, Figure 3 shows the growths of the top six fastest growing and the bottom six slowest growing strains over the 3 days at each of the four temperatures. Further, to visualize the differences in growth between 72 and 24 h, we calculated the difference in growth of each *A. fumigatus* strain at each temperature between day 3 and day 1 (Figure 4). Within each temperature, strains showed high variability in growth difference between the days. Interestingly, except between 35°C and 41°C where no difference in change between growth at day 1 and day 3 was observed in our sample, the remaining pairwise temperature comparisons all showed statistically significant differences (*p*-value < 0.001). Together, our results indicate tremendous variations in growth profiles among strains across the four temperatures and among the 3 days.

### 3.2. *A. fumigatus* strains demonstrate highly variable thermal adaptability to different temperatures

**Figure 5:**
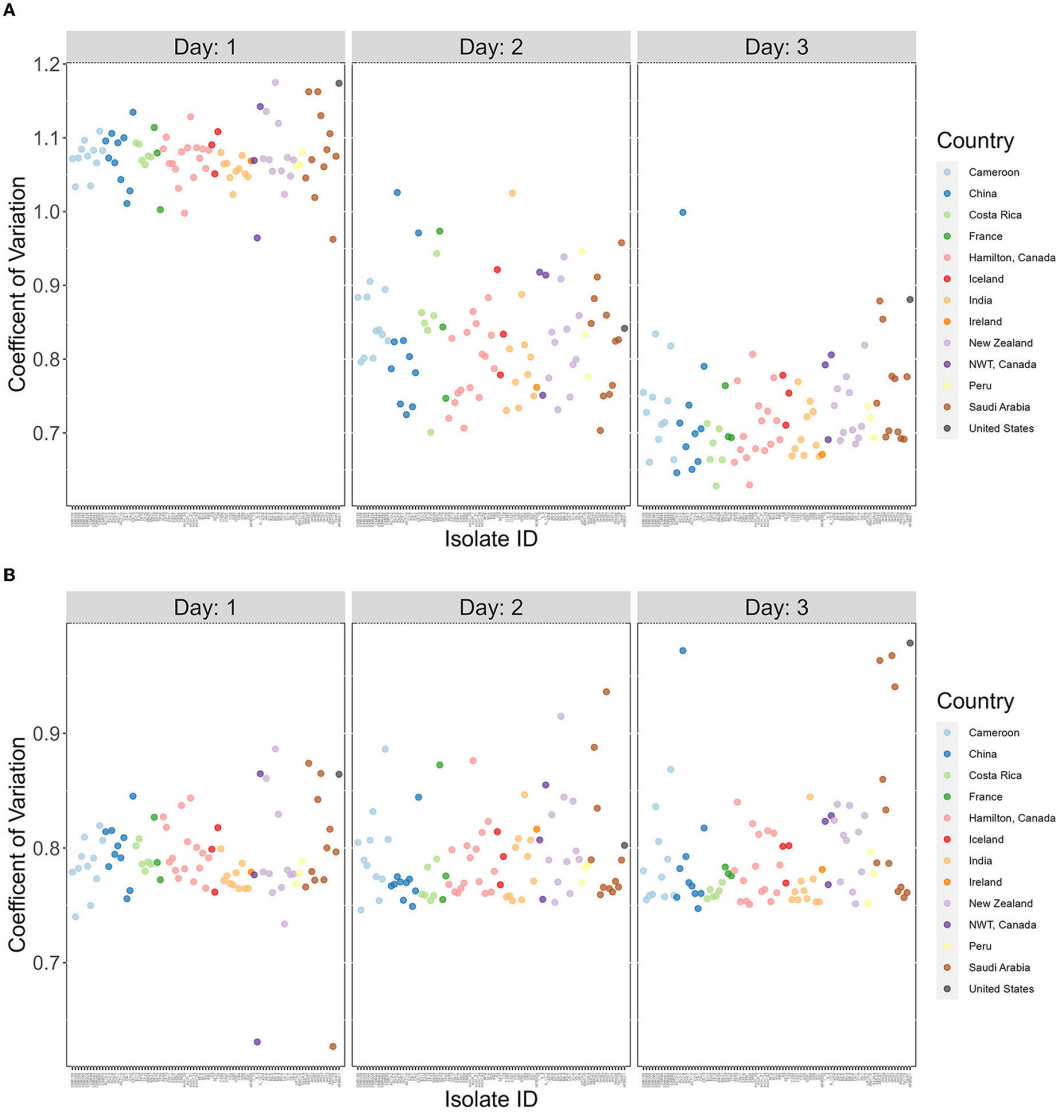
The coefficients of variation are highly variable among *A. fumigatus* strains but decreased over time. X-axis represents 89 *A. fumigatus*. Y-axis represents coefficient of variation (CV) values for each strain. **(A)** CV calculated based on all four temperatures (15°C, 22°C, 35°C, and 41°C). **(B)** CV calculated based on three temperatures (15°C, 35°C, and 41°C).

The broad range of growth between strains seen in Figures 1–4 suggest that there are high variations in growths between strains in their responses to different temperatures. To effectively analyze the broad variations and partition the observed variations to different contributors, we calculated the coefficient of variation (CV), a dimensionless and unitless measure for each strain for each of the 3 days (Figure 5). Our results showed overall highest CV values in Day 1, followed by those in Day 2 and with Day 3 being the lowest. The results suggest big differences in strains' initial responses to different temperatures. However, as time progresses and the strains adapt, the differences in growth among the temperatures decreased. Interestingly, most of the delayed growth occurred at 22°C where limited growths were seen for most strains during the first 24 h but significant growths were observed over the following 48 h at day 2 and day 3 (Figure 5A). Indeed, upon removal of the 22°C data from the dataset, the three-day data showed no significant contribution to differences in CV at the whole sample level (*p*-value = 0.722; Figure 5B). However, obvious variations in CVs among strains were observed (Day 1 range = 0.299, Day 2 range = 0.160, Day 3 range = 0.208).

### 3.3. Geographic origin and the atmospheric temperature at isolation sites have no significant contribution to the high CV between strains

**Figure 6:**
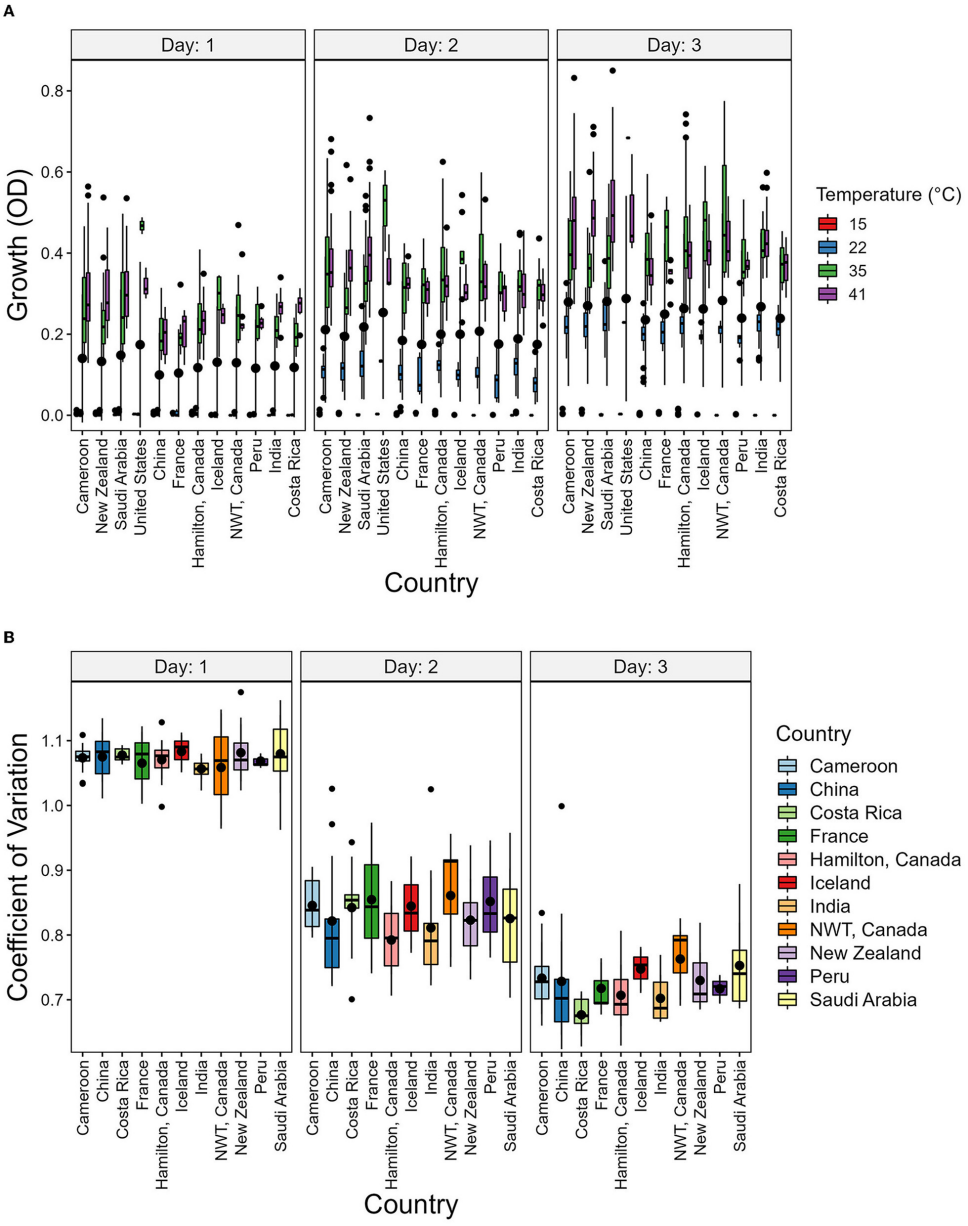
Country of origin has no significant contribution to strain growth and to coefficient of variation (CV). **(A)** Boxplot showing the growth of strains grouped by country and Temperature. **(B)** Boxplot showing the CV of strains grouped by country. The CV was calcuated as the ratio of variance to the mean using the growth values of each temperature.

To determine the potential environmental factors that may contribute to the broad variability in growth and CV among strains, we investigated the impact of geographic origin, the average temperature present at each soil sample site, as well as the range between the highest and lowest temperatures at each sampling site (Table 3 and Figure 6). For geographic origin, strains were grouped by their country of origin. Countries that had < 3 strains were excluded from the analyses. We conducted a mixed ANOVA to determine the contributions of country of origin, temperature, and day post-incubation on strain growth as well as their interaction effects (Table 3). Our ANOVA analyses revealed a significant but relatively minor effect of country of origin on strain growth alone and in its interactions with day post-incubation and temperature. However, though an overall significant contribution based on country of origin was observed, none of the pairwise country comparisons showed significant difference in the growth (Figure 6A) and CV between their strains (Figure 6B).

**Table 3:** Mixed ANOVA on the contributions of country of origin, temperature, and day post incubation and their interactions on the growth of *A. fumigatus* strains.

**Factor**	**DF**	**F**	***p*-value**	**ges**
Country	12	2.773	4 × 10^−3^	0.118
Temperature	3	723.03	4.46 × 10^−85^	0.836
Day	2	1,581.055	1.87 × 10^−102^	0.429
Country : Temperature	39	3.054	8 × 10^−6^	0.206
Country : Day	26	2.079	4 × 10^−3^	0.012
Temperature : Day	6	163.871	8.02 × 10^−61^	0.209
Country : Temperature : Day	72	3.16	3.11 × 10^−8^	0.058

DF, degrees of freedom; F, F statistic; ges, generalized eta squared (effect size values range between 0 to 1 for each).

**Figure 7:**
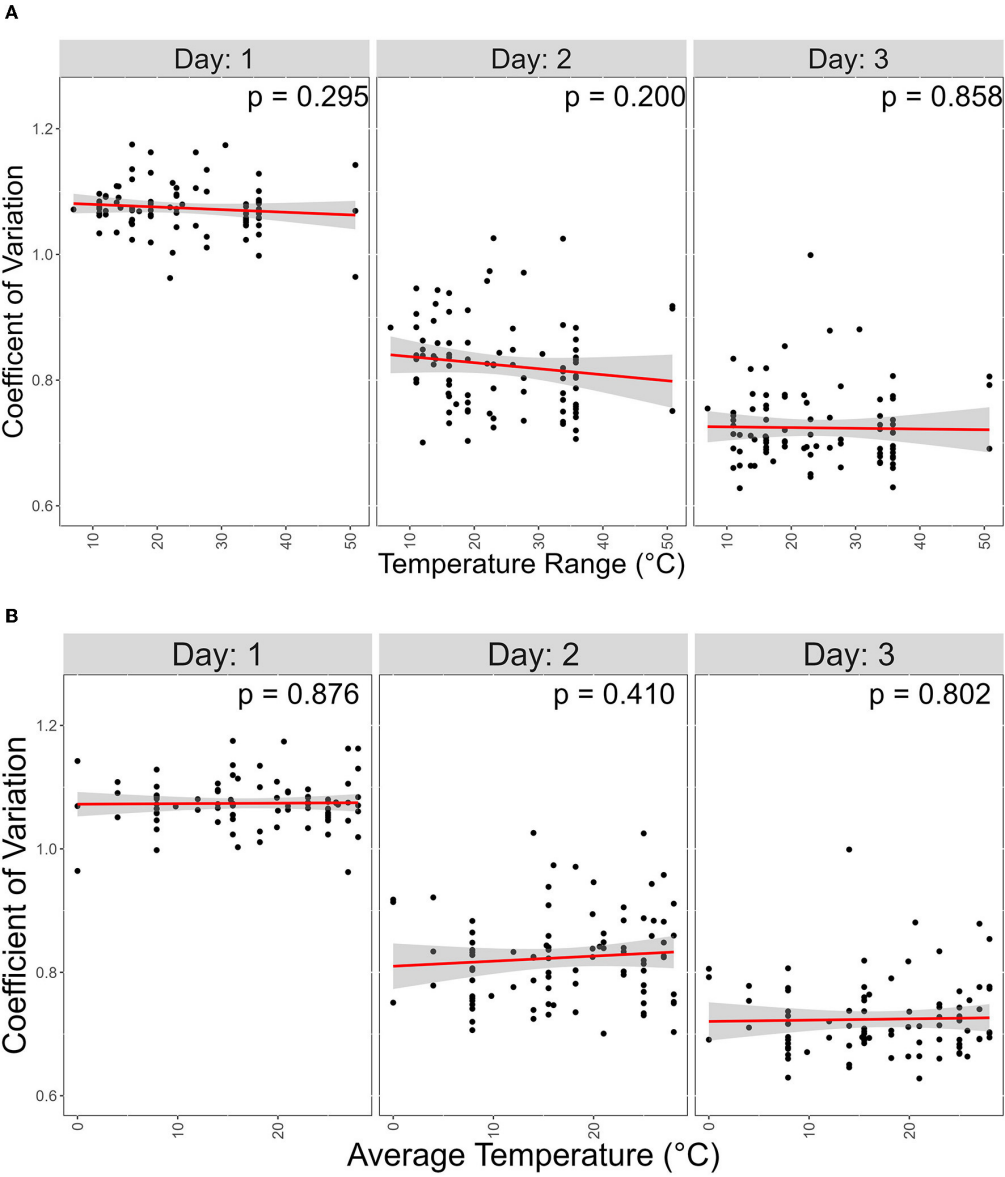
Atmospheric temperature at isolation sites has no significant contribution on coefficient of variation (CV). X-axis represents temperature range; Y-axis represents coefficient variations. Linear regression was used to determine signifcance between the two variables. **(A)** Scater plot of the effect of the Average temperature of each isolation site on CV. **(B)** Scater plot of the effect of the temperature range, which is the difference between the high and lowest average temperature of each isolation site, on CV.

In our data, a low CV represents the ability of a strain to grow similarly at different temperatures. We hypothesized that strains from geographic regions with less varied temperature changes throughout the year will show greater CV than those experiencing more variable temperatures. To investigate this, we obtained atmospheric temperature near the geographic origin of each strain. The contribution of the average temperature and the range between the highest and lowest temperature at each geographic location on CV was analyzed (Figure 7). However, we found no support for this hypothesis.

### 3.4. Strain mating type has minimal contribution to growth and no significant contribution to CV

**Figure 8:**
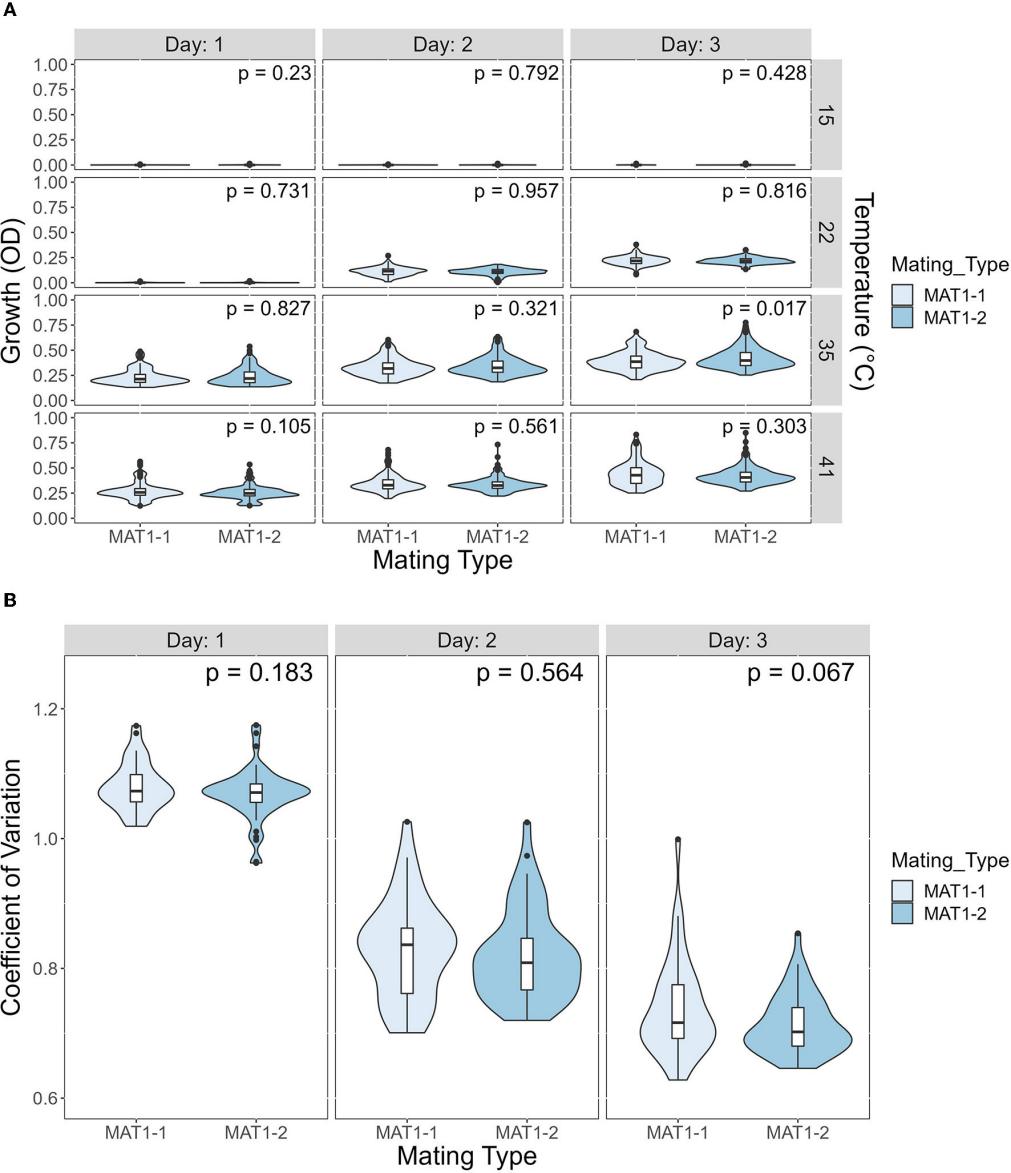
The mating type of *A. fumigatus* strains has a minimal contribution to growth difference and to coefficient of variation (CV). **(A)** Violin plot showing difference in growth between MAT1-1 and MAT1-2 at each temperature on each day. Significance was determined through Mann-Whitney test. **(B)** Violin plot showing difference in CV between MAT1-1 and MAT1-2 at each day. Significance was determined through Student's t-test.

We investigated the potential effects of mating type on growth and CV among temperatures and days post incubation. *A. fumigatus* has two mating type idiomorphs, MAT1-1 and MAT1-2. Mating between strains of opposite idiomorphs are required for sexual reproduction. The details of our analyses results are shown in Figure 8. Overall, our comparisons showed limited difference between the two mating types, in either growth or CV (Figure 8). Except in one comparison, we observed no significant influence of mating type on either growth or CV. The only marginally significant difference observed here were growth at 35°C on day 3 where strains of MAT1-2 overall grew slightly more and had higher CV than strains of MAT1-1.

### 3.5. Genetic distances between strains has no significant association to the pairwise difference in CV

**Figure 9:**
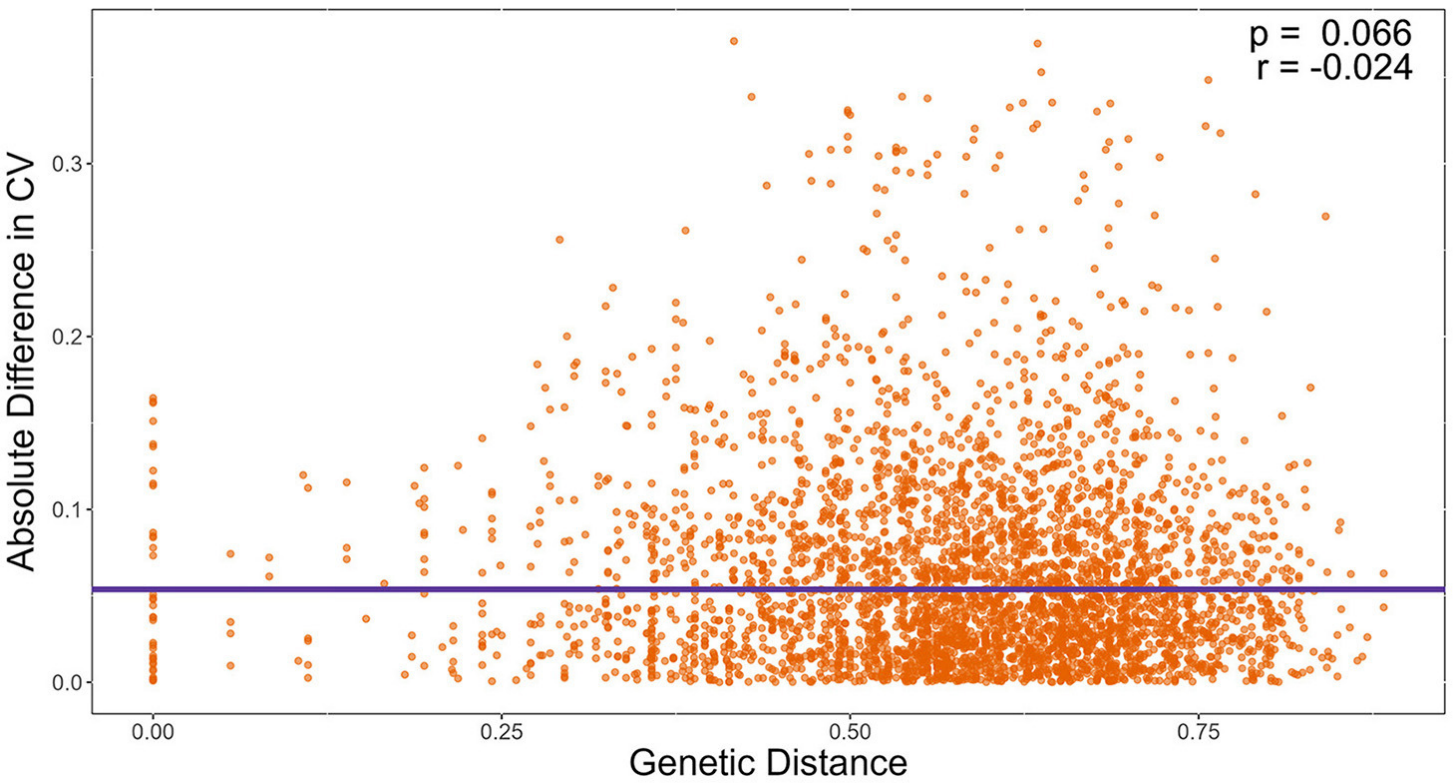
Genetic distance is not significantly correlated with the coefficient of variation (CV) in growths among temperatures. X-axis refers to pairwise genetic distance between strains based on Bruvo's distance. Y-axis refers to pairwise absolute difference in CV on Day 3. Significance was determined through a linear regression model.

**Figure 10:**
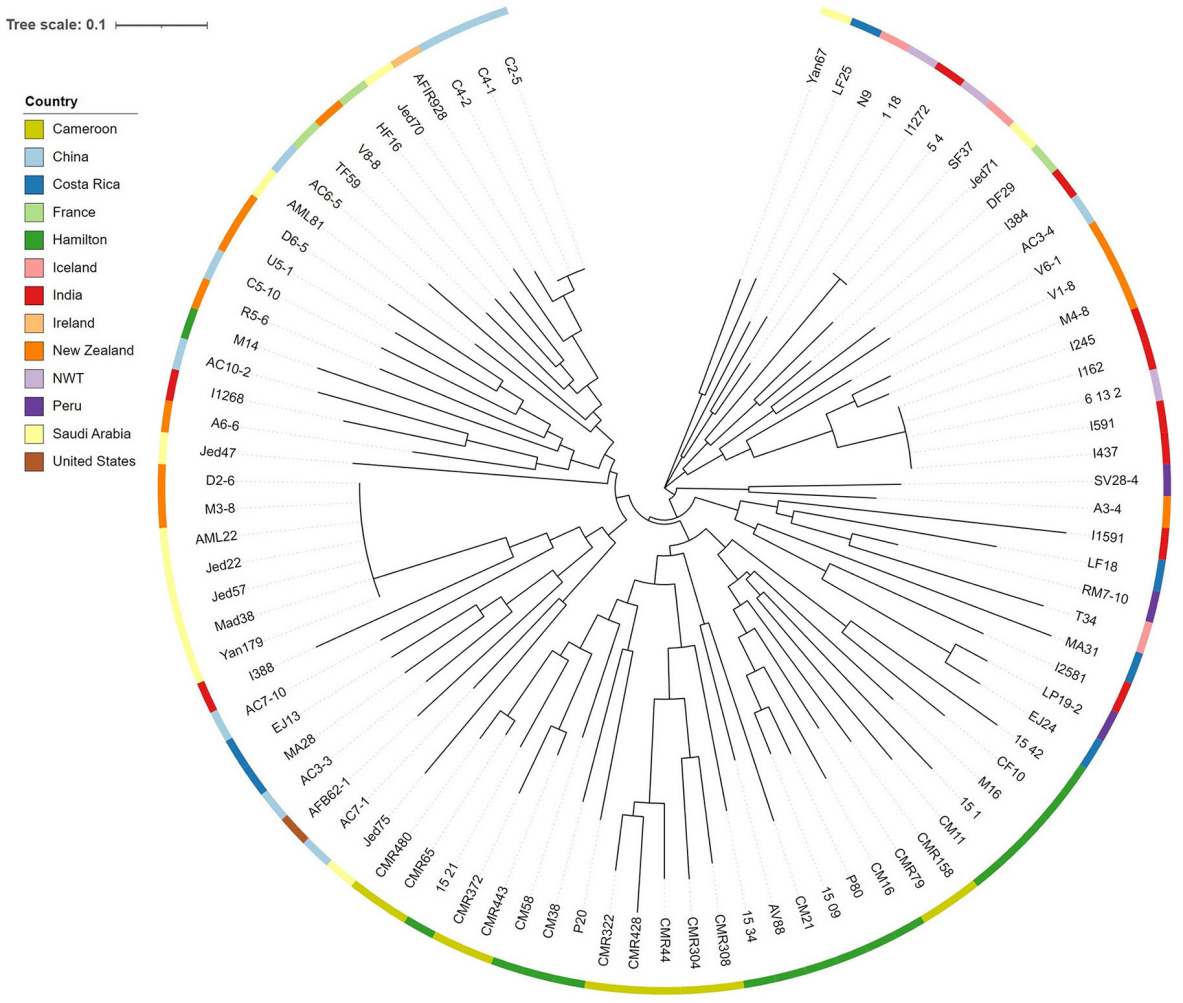
Neighbor joining tree among strains of *A. fumigatus* based on STR genotypes. The phylogram was constructed using the Bruvo's genetic distances at nine STR loci. Color strips at each individual node represent the country of origin.

We also tested the hypothesis that strains with similar STR genotypes would have similar CV. Specifically, we used a linear regression model to determine if the difference in CV between all pairwise combinations of strains grown for 72 h were correlated to their genetic distances. A cubic root transformation on the CV difference was done to achieve a Gaussian distribution among residuals. Our results indicate that the genetic distance between strains was not significantly correlated to CV difference between pairs of strains (Figure 9). Interestingly, the Chinese strain AC3-4 contributed to the top 64 CV difference values. The CV value of strain AC3-4 was 0.999 for day 3, the highest among all strains. Figure 10 shows the relationships among the 89 strains based on their genetic distances inferred from genotypes at nine STR loci.

## 4. Discussion

In our study, we characterized the thermal adaptation of 89 *A. fumigatus* strains from 12 countries representing different climatic areas. After incubation at four temperatures for 3 days, high variation in growth and CV was seen among *A. fumigatus* strains through all 3 days. We then investigated whether genetic and geographic factors contributed to the observed variations in growth and CV. We found that geographic factors such as country of origin, average temperature at isolation site, and the temperature range of the isolation site had no significant contribution to growth or to CV within and among each of the 3 days. Among genetic factors, the MAT1-2 strains showed a slightly higher growth at 35°C on day 3 than the MAT1-1 strains. However, genetic distances as determined based on nine STR loci between strains showed no correlation with their CV differences. Our results highlight the remarkable variation in thermal adaptation displayed among *A. fumigatus* populations regardless of their genotypic similarity and geographic origin. Below we discuss the relevance of our findings to previous studies and their contributions to understanding the evolution and epidemiology of *A. fumigatus* in the future.

Growth differences among strains at different temperatures have been investigated in several fungi, including species of *Aspergillus, Penicillium, Paecilomyces*, and *Metarhizium* (27–31). For filamentous fungi, previous studies measured the radial growth of colonies on solid media at different temperatures as indicators of their growths (32–34). Larger colonies represent higher tolerance to the environmental conditions tested. However, radial growth measures two-dimensional growth *via* colony surface area or colony diameter (32). In comparison, relative optical densities measure three-dimensional growth in liquid media and therefore is likely a more accurate reflection of the total mycelial growth of *A. fumigatus* strains (33). Indeed, liquid culture and OD reading has been used as an indicator for measuring *A. fumigatus* growth for determining their susceptibilities to antifungal drugs (34). In addition, liquid culturing and OD measurements provided us a high throughput method to measure the thermal adaptability of a large number of strains under diverse temperatures through multiple days. However, we note that the liquid culture in the lab can't reflect the dynamic environmental conditions of *A. fumigatus* in nature and that, despite efforts to maintain uniformity, variations among batches and among wells in microtiter plates likely existed that could have contributed to variations in OD among replicates within and among strains.

*Aspergillus fumigatus* is highly thermophilic and has been isolated from soil microenvironments from diverse geographic locations and climates. However, our growth and CV results suggested no significant association between the geographic origins of *A. fumigatus* strains and strain growth patterns at different temperatures. Our results contrast those observed in several other fungal species that showed associations between geographic origin and growth pattern (28, 29, 35–37). For example, *Paecilomyces fumosoroseusis* was shown to have intraspecific variability in growth at different temperatures (28), with increased radial growth at temperatures similar to their climate of origin. The level of yeast pigmentation was also observed to be latitudinally distributed and associated with varying levels of thermal tolerance (37). Dark pigmented yeasts were more commonly found in high latitude regions with lower temperatures whereas light pigmented yeasts were associated with equatorial regions with higher temperatures. Similar to the observed variability in radial growth among strains on solid medium in *P. fumosoroseusis*, we observed tremendous intraspecific variabilities in both growth and CV among our strains. However, in contrast to the growth patterns observed among *P. fumosoroseusis* populations, geographic origin had no significant contribution to the observed variations in our samples. This suggests the individual geographic populations of *A. fumigatus* contain strains with variable levels of adaptability to temperature.

At present, how individual populations of *A. fumigatus* maintain such variabilities is unknown. However, there are three possibilities that may explain the observed variability. In the first, the variabilities in thermal adaptations might be maintained in response to daily and seasonal temperature fluctuations. Within each country and at each site, the daily and seasonal air temperatures can vary widely (38, 39). Second, different ecological niches within a local site can also have different temperature patterns. For example, the inside of a compost pile consisting of decomposing dead organic matter can exceed 50°C while outside of the compost pile the temperature may be <20°C or even lower (40). Third, gene flow could bring strains adapted at temperatures in other sites into new locations (5, 6, 8–10). Indeed, strains with high CV may have originated from geographic regions with limited variability in temperature, such as regions closer to the equator. Gene flow has been observed between distant geographic populations of *A. fumigatus* and such gene flow could contribute to the limited difference among geographic populations in their thermal response profiles (5, 6, 8–10). These three possibilities are not mutually exclusive and all three could have contributed to the observed variabilities, with potentially different contributions to different geographic populations. Greater sampling of diverse ecological niches within specific geographic regions combined with temperature-based experimental evolution studies could help determine the extent of their contributions to *A. fumigatus* growth variations at different temperatures (1, 41).

The genetic mechanisms underlying thermal tolerance have been examined in several fungal species. Though some genes and mutations can have major effects on thermal tolerance (41), most studies have shown that thermotolerance is a polyphyletic trait that emerged multiple times throughout the fungal phylogeny (30, 42). Our BSH results suggested genetic differences between strains contributed to variations in thermal growth profiles among strains of *A. fumigatus*. Therefore, we tested the contribution of strain mating type and STR genotype on strain thermal adaptivity. Our results suggested mating type had minimal effect on growth and no effect on CV, where the only significant association was for day 3 at 35°C. For ascomycete fungi, mating type idiomorphs predominantly function as transcription factors that regulate the sexual cycle (43). However, in some fungi, genetic pathways influenced by mating type intersected with other pathways including those for conidiation, stress response, and pathogenicity (44, 45). In *A. fumigatus*, a temperature around 65°C is required to germinate sexual ascospores (46). Sexual reproduction within composting plant-waste material has recently been observed between *A. fumigatus* strains, where temperature above 65°C readily occur (47). Given that the sexual reproductive pathways intersect with many other pathways and sexual reproduction requires elevated temperatures to occur in *A. fumigatus*, there may exist a possible link between sexual pathways and thermal adaptability. Although no signification correlation between mating type and thermal adaptability was observed in our study population, an association may exist in some strains. An example of this potential association was the Chinese strain AC3-4. In our study, AC3-4 showed the highest CV and contributed to the highest CV differences between strains. Interestingly, our previous work showed that strain AC3-4 was among the most fertile in the samples we tested (48). Given the data we presented here, it's templating to speculate that there may be an association between high mating ability and high CV for growth at different temperatures for a subset of strains within many geographic populations of *A. fumigatus*. Interestingly, another supermater strain AFB62-1 also had a similarly high CV value (20, 48). Further investigation is required to identify if and how mating ability influences thermal adaptability.

Interestingly, the genetic distance between strains estimated through nine STR markers showed no relationship to their CV differences. This lack of association suggests that the STR markers were indeed neutral with regard to thermal growth profile differences among strains and therefore they were unable to explain the observed variance in CV among strains. Frequent recombination between strains within and between many *A. fumigatus* populations would erode any linkage disequilibrium between the nine STR markers and genes associated with growth at different temperatures (5, 6, 8). Indeed, a recent study by Auxier et al. found that *A. fumigatus* has the highest number of crossovers during meiosis among eukaryotic species, approximately 29 crossovers per chromosome during each meiosis (49). This high recombination rate will allow genetically unique migrants, upon entering a local population, to quickly acquire and/or spread genes that promote adaptation to the native climate. While the neutrality of the nine STR markers are ideal for characterizing *A. fumigatus* population structure, further research is required to genetically explain the observed variance in thermal adaptation in our *A. fumigatus* population. Multiple *A. fumigatus* genetic pathways are known to be upregulated during thermal stress (38–41). For example, the nucleolar protein CgrA is upregulated in *A. fumigatus* and is thought to increase the production of proteins related to heat response (50). More recently, proteins that regulate aspects of the cell wall integrity pathway have been shown to provide resistance to heat shock, such as protein chaperone HSP90 and the heat shock transcription factor HsfA (51, 52). In another model filamentous fungus *Neurospora crassa*, mutants of calcium/calmodulin-dependent kinases were observed to have reduced thermotolerance (53). In future studies, a GWAS analysis may locate previously identified and/or novel gene regions under selection for thermotolerance and provide candidate genes for gene expression quantification that may explain the observed variance in growth among strains. Additionally, a proteomic analysis of the strains may further elucidate the impact of thermal stresses on the *A. fumigatus* proteome (54).

## 5. Conclusions

Rising global temperatures due to climate change will promote the expansion of microbial pathogens toward higher and lower latitudes, as well as cause the emergence of novel pathogenic species (1). More research is required to better understand the impact that rising global temperatures may have on microbial populations. Populations of *A. fumigatus* have broad global distributions, and therefore have the potential to adapt to a wide range of climatic temperatures present in these diverse ecological niches. Our findings of high variance in growth among strains across temperatures irrespective of geographic origin and genetic distance suggest the extreme capacity of local populations of *A. fumigatus* to adapt to the changing climate and global warming. Coupled with the high speed at which *A. fumigatus* disperse, strains and populations with highly adaptive mutations to thermal stresses could spread rapidly and be integrated into local populations across the globe (18, 49). In additional to rising global temperatures, climate change will alter other abiotic factors within soil environmental, such as water availability and solute concentrations that fungi will need to adapt to in the coming years (27). Therefore, further investigation into the adaptability of *A. fumigatus* strains to abiotic stressors will provide insights on the remarkable ability of *A. fumigatus* populations to acquire and spread highly fit genotypes, including those that provide resistance to antifungal drugs.

## Data availability statement

The datasets presented in this study can be found in online repositories. The names of the repository/repositories and accession number(s) can be found in the article/Supplementary material.

## Author contributions

GK led the study, contributed to the data collection, analyses, interpretation, and manuscript drafting. EH contributed to the data collection and manuscript writing and edits. JX conceptualized the study design, contributed to data interpretation, and manuscript writing and finalization. All authors read and approved the final manuscript.

## Supplementary material

Supplementary Table 1 Strain genotype, mating type, and geographic information for all 89 strains analyzed in this study.

Supplementary Figure 1 Reaction norm plots showing strain × temperature interactions in the growth profiles of 89 strains after three days of incubation across four temperatures separated by country. NWT, Northwest Territories, Canada.
